# Research Progress of Extracellular Vesicles-Loaded Microneedle Technology

**DOI:** 10.3390/pharmaceutics16030326

**Published:** 2024-02-26

**Authors:** Xue Wang, Wei Cheng, Jiandong Su

**Affiliations:** 1 Department of Burn and Plastic Surgery, Suzhou Hospital Affiliated to Nanjing Medical University, Suzhou 215000, China; 2022121959@stu.njmu.edu.cn; 2 Jiangsu Key Laboratory of Thin Films, School of Physical Science and Technology, Soochow University, Suzhou 215006, China; 20234008011@stu.suda.edu.cn

**Keywords:** microneedle, extracellular vesicles, exosomes, drug delivery, diagnosis evaluation, prognostic monitoring

## Abstract

Microneedles (MNs), renowned for their painless and minimally invasive qualities, exhibit significant potential for facilitating effective drug delivery, vaccination, and targeted sample extraction. Extracellular vesicles (EVs), serving as cargo for MNs, are naturally occurring nanovesicles secreted by cells and characterized by novel biomarkers, low immunogenicity, and cell-source-specific traits. MNs prove instrumental in extracting EVs from the sample fluid, thereby facilitating a promising diagnostic and prognostic tool. To harness the therapeutic potential of EVs in tissue repair, MNs with sustained delivery of EVs leverage micron-sized channels to enhance targeted site concentration, demonstrating efficacy in treating various diseases, such as Achillea tendinopathy, hair loss, spinal cord injury, and diabetic ulcers. EV-loaded MNs emerge as a promising platform for repair applications of skin, cardiac, tendon, hair, and spinal cord tissues. This review commences with an overview of MNs, subsequently delving into the role of EVs as cargo for MNs. The paper then synthesizes the latest advancements in the use of EV-loaded MNs for tissue regenerative repair, extending to research progress in extracting EVs from MNs for disease diagnosis and prognostic evaluations. It aims to offer valuable insights and forecast future research trajectories with the hope of inspiring innovative ideas among researchers in this field.

## 1. Introduction

Microneedles (MNs) are microscopic needle-like structures, typically fashioned in a pyramid shape, with heights ranging from 150 to 1500 μm, widths between 50 and 250 μm, and tip thickness between 1 and 25 μm [1,2]. They are specifically engineered to penetrate the skin’s stratum corneum, creating micron-sized mechanical channels on the surface, thereby contributing to local effective drug concentration and facilitating various medical applications [3]. In routine transdermal drug delivery, MNs are distinguished for painless puncture [4], scarless healing [5], and a few gastrointestinal degradations and hepatic first-pass metabolisms [6]. In addition, recent advancements have seen the development of MNs for targeted drug delivery to various non-cutaneous organs and tissues, such as facilitating drug entry into the oral mucosa [7], eyeballs [8,9], cardiovascular tissues [10,11], spinal cord [12,13], scalp hair follicles [14], endometrium [15], etc. Using MNs to deliver drugs to different anatomical sites needs to include sensitive biosensor design and miniature flexible and biocompatible electronics in the assessment [12]. MNs can also be integrated with multiple sensors for detecting skin interstitial fluid (ISF), consistently monitoring ion concentrations, glucose, uric acid, insulin, and serotonin, providing a novel approach for disease diagnosis and prognosis monitoring [16,17,18]. Considering factors such as material composition, shape, structure, mechanical strength, and biodegradability, MNs can be custom-designed for various therapeutic applications [19]. For instance, MNs’ dressings equipped with multiple drug loads and enhanced mechanical strength not only minimize mechanical damage to non-healing wounds but also significantly expedite the wound-healing process [20]. The programmed core–shell structure of MNs can be used to regulate the inflammatory microenvironment according to the healing stages of chronic wounds by releasing drugs in a sequential response [21]. Extracellular vesicles (EVs), not only as messengers of cell-to-cell interstitial signaling exchange but also as carriers of multifarious bioactive molecules, nutrients, and garbage, exert various cellular life activities depending on different cellular origins [22,23]. A specialized type of extracellular vesicles, called matrix vesicles, can induce mineral formation in bone tissues due to their abundant calcium and phosphorus content [24]. The properties and cargo-carrying of EVs are decided by primitive cells [22]. For instance, depending on various cell types, EVs carry cell-type-specific proteins displaying specific fates and functions [25]. However, challenges in their application arise, as EVs repeatedly administered through intravenous injection lead to accelerated clearance in the circulatory system [26]. Additionally, EVs applied locally often face degradation and a consequent loss of their therapeutic activity [27]. To mitigate the accumulation of EVs in non-target organs and prevent their premature clearance, EVs are engineered to be modified by targeting their membrane components or contents [28,29]. It is also necessary to develop continuous delivery of EVs [30]. Thus, the availability of MNs for delivery has also been proposed as a relatively alternative method [31]. Researchers have recently explored a range of applications utilizing MNs for the delivery of EVs to intradermal or other non-cutaneous tissues, as well as employing MNs for the detection of EVs in specific bodily or interstitial fluids [32,33,34,35,36]. These advancements underscore the considerable potential of MN-mediated EVs in therapeutic and diagnostic applications for prognostic purposes in clinical settings [37].

This paper begins by presenting the current state of MN technology, delving into the sources, extraction methods, and status of applications involving loaded EVs. Subsequently, a comprehensive literature review was conducted on MNs and EVs from the past decade, utilizing databases such as PubMed, OVID, and Web of Science. The focus is on highlighting the most recent advancements in MNs loaded with EVs for both percutaneous and non-percutaneous delivery. Moreover, the paper outlines effective strategies for the extraction of EVs using MNs, encompassing diagnostic prognosis as well as purification and isolation techniques. Finally, it discusses the limitations encountered in the application of EV-loaded MNs and anticipates future research directions in this burgeoning field.

## 2. Types and Materials of Microneedles

**Figure 1:**
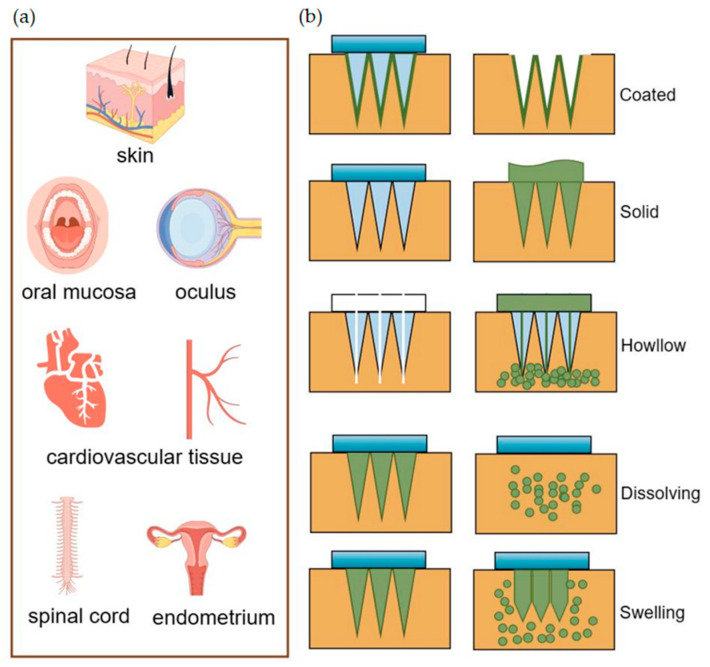
(**a**) MNs deliver drugs for transdermal and non-transdermal applications. (**b**) Drug diffusion diagram of different types of MNs.

The skin, serving as a vital protective barrier for the human body, often impedes the penetration of effective drug concentrations into the deeper tissues of wounds, particularly in cases of chronic bacterial or fungal infections caused by external injuries or immunosuppression [38,39,40]. MNs, as an innovative tool in the realm of drug delivery, are designed to inject drugs locally into the epidermis, superficial dermis, or deep dermis by penetrating the skin’s stratum corneum [41]. Research indicates that the insertion of MNs, particularly those under 1000 μm in size, avoids contact with nerve tissue and dermal blood vessels [42]. This feature allows for the achievement of painless injections, making MNs a highly effective and patient-friendly option for localized drugs [43]. In addition, MNs can be administered with less discomfort than conventional injections, increasing patient compliance, fastening puncture area healing, and avoiding the first-pass metabolism of orally administered drugs [44,45,46]. Some MNs are prepared using specially designed materials, such as PN-Si, chitosan metal nanocomposites, etc., which are naturally antimicrobial, and thus, MNs themselves can be used as therapeutic agents to promote tissue repair [47,48,49]. In addition, MN-based biosensors effectively capture dermal interstitial fluid (ISF) [50] and have been shown in preclinical experiments to be effective in monitoring blood glucose [51], electrolyte levels [52], Ph level [53], and biomarkers such as epidermal growth factor receptor 2 [54], carcinoembryonic antigen [55], cystatin C, etc. [56]. According to different drug release mechanisms, MNs are mainly categorized as coated, solid, hollow, dissolved, and soluble [57]. As shown in Figure 1, they have a wide range of tissue administrations.

The resealing time of human skin following MN treatment varies from 3 to 40 h, which can effectively enhance skin permeability for a viable duration, thereby facilitating improved drug absorption [58]. Coated MNs are applied by puncturing the skin and delivering the drug coated on their surface to the puncture site. However, an MN patch with a size of about 10–20 cm^2^ can only be coated with up to 1 mg of the drug, which constrains its ability to deliver effective drug concentrations for certain applications [59,60]. Enhancing the coating process to achieve a uniform coating can increase the maximum drug-loading dose of MNs without compromising the drug’s activity [61]. This improvement offers promising prospects for the large-scale production and utilization of MN patches [62,63]. The application of solid MNs involves a two-step process: initially, they pierce the skin to create a channel, followed by the application of a topical preparation [64]. However, solid MNs, along with hollow MNs, are susceptible to needle tip breakage, with the potential for the broken needle fragments to remain embedded in the skin tissue, posing biocompatibility issues [59,65]. Research indicates that the use of solid MN rollers to puncture the skin, followed by the application of EVs derived from human umbilical cord mesenchymal stem cells (HUC-MSCs), can effectively alleviate the symptoms of melasma patients, offering an enhanced experience for the patient [66]. Hollow MNs are similar in principle to the routine use of syringes and have clinical advantages in simple steps for doctors to follow [67]. As the name suggests, hollow MNs have holes in the tip and utilize a mechanism of “poke-and-flow” to deliver the drug to the skin tissue, which allows for high-dose administration compared to other types of MNs. However, a notable disadvantage is that these needles possess relatively weak mechanical strength, making them prone to breakage, and the rate of drug delivery can lead to obstructions within the lumen [3,68]. Dissolving MNs dissolve after insertion into the skin, and the drug is released with hydrolyzed or enzymatically dissolved polymers, which sustainably maintain the drug concentration and show promise toward wounds requiring prolonged healing [69]. Additionally, Han and colleagues have employed digital light processing and 3D printing techniques to fabricate polymeric MNs with barbed tips aimed at enhancing tissue adhesion and maintaining the duration of drug release [70]. The swelling MNs consist of crosslinked hydrogels that swell upon insertion into the skin by absorbing water, and the hydrogels have the ability to adapt to and mimic mechanical changes over time with a high drug loading capacity and adjustable drug release rate, overcoming the limitations of conventional MNs [71,72]. You and their team have demonstrated that the delivery of human dermal fibroblast-derived EVs using hyaluronic acid MN patches effectively reduces skin wrinkles [34]. Additionally, the loading of these patches with EVs-encapsulated collagen mRNAs leads to sustained collagen implantation in the dermis, further enhancing the improvement of skin wrinkles [34].

Materials used to prepare MNs include silicon [73], metal [6], ceramics [74], silica glass [2], silk proteins [75], polymers [76], etc., which have been summarized in detail in many reviews [3,27,77]. Polymer MNs have received more attention in recent years, and here, we focus on new advances in the preparation of MNs from this material.

A diverse range of polymers is utilized in the fabrication of MNs, commonly encompassing soluble polymers such as sodium hyaluronate, sodium carboxymethylcellulose, polyvinyl alcohol, polyvinylpyrrolidone, and degradable polymers such as polylactic acid, chitosan, poly or polylactic acid-hydroxyacetic acid copolymer, etc. [78]. In comparison to MNs made from other materials, the mechanical properties and drug release characteristics of polymer-based MNs can be tailored by modifying factors such as the cross-linking density, concentration, molecular weight, and charge properties of the polymers [79]. Soluble MNs are predominantly used in in vivo settings and recognized for their capability to quickly release drugs or vaccines within the body [80]. An example of this is noted in a study by Ito et al., where it was observed that using dextrin as a matrix material for MNs facilitated the release of almost the entire quantity of formulated insulin within just one hour [81,82]. Designing MNs with a dual-layer structure, comprising a matrix layer and a backing layer, enhances their drug delivery capabilities [83]. The matrix layer allows for rapid administration to achieve the effective dose, while the backing layer continuously replenishes the drug [84]. This design supports prolonged, effective drug release, contributing to a stable therapeutic environment [85]. Patricia G-V and colleagues developed dissolvable MNs for treating neonatal sepsis, utilizing sodium hyaluronate and polyvinylpyrrolidone as the materials for the matrix and the backing layers of the MNs, respectively [86]. In this study, gentamicin was initially administered from the needle’s tip, followed by the sustained release of the antibiotic from the backing layer through the created pores, ensuring continuous drug delivery [86]. Zhang et al., prepared hydrogel-structured modular MNs for multiple delivery of antibiotics, IL-4, and TGF-β for the treatment of periodontitis, in which the basement membrane of the MNs rapidly dissolved to release the antibiotics, and silica particles and biodegradable nanoparticles acted as the carriers for the drugs and cytokines in the hydrogel structure of the MNs, slowly releasing the encapsulated contents [87]. Similarly, the encapsulation of successfully isolated EVs derived from human adipose stem cells into the tips of MNs crafted from polymeric materials has been shown not only to preserve their molecular activity over an extended period but also to effectively regulate the release of these EVs [88].

Hydrogels are three-dimensional porous polymer networks prepared by the physical or chemical cross-linking of hydrophilic molecules, and one of the most commonly used polymers is poly methyl vinyl ether-co-maleic acid cross-linked with polyethylene glycol [89]. The hydrogels undergo swelling upon insertion into the skin without dissolving. Further, they have chemical and mechanical properties similar to those of human tissues, are biocompatible, soft, and stretchable, and can be self-healing [90,91,92,93,94]. Hydrogels find extensive applications in fields such as tissue engineering, sensor fabrication, drug delivery, and biological research. It is capable of providing an intelligent drug delivery system that responds to various types of stimuli, such as temperature [95,96,97], light [98], Ph [99,100], glucose concentration [101], active oxygen species [102], etc. Compared with other types of MNs, hydrogel MNs have the outstanding advantages of significantly higher drug loading and intelligent control of drug release rate [103,104]. Guo et al., prepared glucose-responsive hydrogel-based MN dressings with strong adhesion to diabetic wounds and responsive release of insulin to different concentrations of glucose, accelerating the diabetic wound healing process, reducing inflammatory response, and realizing intelligent drug release [105]. In addition, utilizing the water absorption and swelling characteristics, Razzaghi et al., designed 3D-printed hydrogel MNs arrays of polyethylene glycol diacrylate material integrated with multiple sensors, which allowed for the extraction of biomarkers from interstitial fluids and colorimetric diagnostic assays in a few minutes [106]. The multifunctionality and unique properties of hydrogels make them ideal systems for biomedical applications, and the preparation of soft dressings from hydrogel MNs combined with EVs is currently a research hotspot for wound repair [107]. While polymers and hydrogel materials demonstrate promising application prospects, current research often transcends the use of a single, simple polymer for MN fabrication [108]. Instead, researchers are exploring related modifications or combinations of various materials to achieve enhanced application outcomes [109]. For instance, the development of multifunctional MNs based on a magnesium metal–organic framework combined with hydrogel has shown significantly improved wound healing in diabetic mice [110]. Soluble MNs were prepared by integrating textured polysaccharides into diphenyl carbonate cross-linked cyclodextrin metal–organic frameworks, which exhibited higher mechanical strength and better physical stability than MNs made of single hyaluronic acid [111].

## 3. Loaded Cargos of Microneedles

On the one hand, MNs can effortlessly puncture tissues, change the local stress environment, induce skin collagen deposition and reorganization, and provide natural mechanical stimulation for tissue regeneration and wound repair [112,113]. On the other hand, MNs fully demonstrate their potential in diabetes, superficial tumors, Alzheimer’s disease, infected wounds, contraception, and other therapeutically diverse applications by carrying a wide range of drugs, including small or large molecules, vaccines, nucleic acids, nanoparticles, EVs, and cells, among others [12,59,114,115,116].

Given that this review concentrates on the application of EV-loaded MNs, our focus will be on the isolation methods and applications of EVs. EVs were initially identified in mature reticulocytes and peripheral blood platelets as globular membrane vesicles, distinct from the small cellular fragments shed by dying and damaged cells [117,118]. Almost simultaneously, matrix vesicles (MVs) were observed as electron-dense ‘leaf-like’ particles with ‘needle-like’ projections within an ossifying cartilaginous matrix [119]. Thanks to the more widespread physiological contributions of EVs, advancements in our understanding of MVs have occurred mostly in parallel with associated developments in EV biology [120]. A notable study shows that EVs from MSCs presented a clinical benefit to patients suffering from Menière’s disease by acting as a local adjuvant treatment, which is of great significance for EVs to become clinical therapeutic agents [121]. Extensive research has revealed that EVs are abundantly present in various biological fluids, including plasma, intercellular fluid, cerebrospinal fluid, urine, sperm, bile, synovial fluid, saliva, and breast milk, as well as in malignant fluids in pathological conditions, effectively facilitating cellular communication by transmitting signals [122,123]. Of note, EVs are considered to be one of the structural and functional components of the extracellular matrix [23]. EVs are now characterized as bilipid membrane structures carrying cell-specific nucleic acids, proteins, lipids, and other bioactive molecules. These molecules bind specifically to target cells, altering the structure and function of the recipient cell [124]. Not only EVs but also non-vesicular nanoparticles can carry nucleic acids, proteins, and other bioactive molecules. Based on their size, biogenesis, secretion mechanisms, surface markers, and physiological functions, EVs are classically categorized into microvesicles, apoptotic vesicles, and exosomes (exos) [125]. Recent studies have discovered new EVs, such as autophagic EVs, stressed EVs, and matrix vesicles [126]. Additional special types of EVs include membrane granules, exos-like vesicles, neutrophil-derived microvesicles, and prostasomes [127,128]. High-resolution imaging and tracking of EVs are challenging, and specific subgroups are difficult to identify with biomarkers with 100% accuracy, leading to the potential misinterpretation of the overall effect of EVs as a heterogeneous presentation of a subgroup [125,129]. Despite these challenges, EVs have leveraged existing isolation techniques, demonstrating significant therapeutic and companion diagnostic potential. Here, we offer a detailed description of their isolation methods prior to microneedling and their applications.

### 3.1. Isolation Methods of EVs

Gámez-Valero et al., emphasized the importance of selecting appropriate EV isolation methods tailored to the specific characteristics of EVs [130]. Most of the current isolation methods for EVs have been developed based on the biophysical principles of size separation, immunoaffinity capture, and density precipitation and can be broadly categorized into four groups with their notable properties summarized [131] (listed in Table 1). Among these, ultracentrifugation, the most commonly employed technique for EV isolation, is straightforward but fails to completely segregate EVs from other vesicular structures or proteins [132]. Moreover, high-speed centrifugation may compromise the integrity and activity of EVs [133]. Ultrafiltration-based EV isolation can shorten processing time and obtain a comparable yield to the ultracentrifugation method [112]. Several commercially developed methods for EV enrichment and isolation leverage a combination of separation techniques for efficient retrievals, such as simple dilution filtration through ligand-based EV affinity purification column chromatography, followed by centrifugal filters to procure purified platelet-derived EVs [134]. Based on polymer precipitation and aqueous two-phase system separation, the polymer precipitation method creates a hydrophobic microenvironment for obtaining EVs, which is faster and easier than ultracentrifugation methods [135]. Additionally, Nwokwu et al., employed the immunoaffinity principle for adhering EVs to porous MN structures and then isolated EVs directly from biological fluids [136]. This approach offers a broad dynamic range of temperature and incubation parameters, enhancing flexibility for laboratory and clinical applications [136]. Although the immunoaffinity method can distinguish between subgroups of vesicles, it only allows the nonspecific isolation of the EVs [137]. Recent advances in microfluidic technologies show promise for clinical utility due to their low yield and high sensitivity, particularly in diagnostic applications [138]. However, standardized protocols for EV isolation and identification techniques are lacking. Isolation techniques that enable access to high-quality, large quantities of EVs at a low cost are crucial for further research to facilitate mass production and expand medical applications.

### 3.2. Applications of Extracellular Vesicles (Preclinical Studies and Clinical Trials)

As research on EVs intensifies, there is growing interest in utilizing EVs as sub-cellular therapies for various diseases and as prognostic markers in disease diagnosis [147,148]. To date, the majority of EV products have not yet received approval for commercial production or clinical use due to challenges in quality control and safety concerns, though clinical and preclinical studies are on the rise annually [149]. Compared to the direct application of stem cells for disease treatment, EVs offer numerous advantages, such as high biosafety, ease of storage and transportation, rapid efficiency, and a broad range of sources [150]. The heterogeneity of EVs is attributed to the diversity of target cell receptor phenotypes and the variety of source tissues or organs. However, due to constraints related to cell activity, stimulation conditions, isolation techniques, and storage conditions, even EVs secreted by the same type of cell may exhibit significant functional differences [151,152].

EVs perform physiological functions akin to their cells of origin but exhibit relatively low immunogenicity and tumorigenicity risks and may traverse the blood–brain barrier, offering a highly effective cell-free therapy [153,154]. In fact, much of the evidence for EV transfer across the blood–brain barrier is indirect. A study reported that EVs administered intravenously in mice were detectable in cerebrospinal fluid [26]. Studies have demonstrated that EVs play roles in prostate cancer therapy and tissue engineering by being incorporated into biomaterials and bone regeneration [155,156,157]. On one hand, hepatocellular carcinoma cell-derived EVs can promote tumor formation and metastasis by inducing changes in surrounding non-cancerous cells [158]. On the other hand, they can also play an anti-cancer role by activating immune responses through the presentation of neoantigens and/or MHC–peptide complexes [159]. EVs play diverse or sequential roles in the cancer process, indicating the need for further research into their mechanisms of action for restorative purposes [160]. Yuan et al., demonstrated that adipose stem cells (ADSCs)-EV promote fibrosis and dermal regeneration in the early stages of wound healing and reduce scar formation in later stages [150]. Free EVs, lacking a controlled release mechanism, can be utilized in scaffolds not only to replace damaged or non-functional tissues but also to enhance the bioavailability of EVs at the site of action [161]. EVs are also widely used in tissue regeneration, such as improving myocardial fibrosis in arrhythmogenic cardiomyopathy [162], regenerating the retina [163], repairing damaged liver [164], improving nerve regeneration in neurological disorders [165], and promoting bone and cartilage regeneration and differentiation [166] (all proven effective in clinical or animal experiments). Currently, MSC-derived EVs have become a hot research topic and are widely used as ideal drugs or carriers for drug delivery [167]. Many studies have shown that MSC-derived EVs can promote wound healing, normalize skin structure, and reduce scar formation in various conditions like burn wounds, diabetic wounds, systemic sclerosis, radiation dermatitis, and excisional wounds [168,169]. Moreover, modifying parental MSCs or pretreatment with certain components can enhance the therapeutic effects of MSC-derived EVs. For instance, lipopolysaccharide-pretreated EVs showed a better ability to regulate macrophage homeostasis by upregulating the expression of anti-inflammatory cytokines and promoting the activation of M2 macrophages compared to untreated MSC-derived EVs [170]. This modification or pretreatment of EVs opens new horizons for further exploration of their mechanisms of action.

EVs emerging as effective biomarkers are capable of delivering specific proteins, lipids, messenger RNAs, non-coding RNAs, and other bioactive compounds [171,172]. Therefore, at present, EVs can only serve as a valid reference in disease diagnosis, and further large-scale studies are required to establish them as a diagnostic standard. A meta-analysis incorporating the detection of positive EV biomarkers of exoDNA KRASmut, ExmiR 451 a, ExmiR 200 b, ExmiR 222, and so on in 634 patients indicated that the presence of positive EV biomarkers in the blood varied in correlation with mortality at different stages of pancreatic ductal adenocarcinoma treatment [173]. This illustrates the effectiveness of EVs in disease prognosis, as endothelium-derived EVs expressing CD146 and CD105 were significantly predictive of overall survival in metastatic colorectal cancer [174]. Additionally, investigators have found significant variability in EVs between patients with castration-resistant prostate cancer who are sensitive or resistant to docetaxel, making it a valuable source of prognostic biomarkers [175].

## 4. Extracellular Vesicles-Loaded Microneedles: A Promising Direction

EVs are administered through various methods, including intravenous injection, intraperitoneal injection, local injection, and nebulized inhalation [176,177]. While intravenous injection is less commonly utilized, EVs have demonstrated a homing ability akin to that of parental cells and can efficiently accumulate in wound areas to aid in the healing process [178]. However, they are rapidly degraded in the systemic circulation [178]. Utilizing MNs for the target delivery of EVs to intradermal or other non-cutaneous tissues facilitates sustaining the local effective drug concentration of trauma [179]. In contrast, nebulized inhalation of EVs allows direct delivery to the fine bronchioles and alveoli, optimizing drug concentration for pulmonary applications [177]. Studies have shown that fluorescent-labeled human adipose-derived MSC-EVs reached peak concentrations in the lungs of mice with severe pneumonia 24 h post-nebulization, with a gradual decline over 28 days [180]. Local injection, though direct, is hindered by secondary wound injury and fluid flow in the body, limiting EV penetration and retention at the wound site, thus diminishing treatment efficacy [13]. Cao et al., developed a photoaging model on hairless mice to compare the effects of MN rollers alone versus a combination of topical application of ADSCs aqueous EVs [32]. Findings revealed that the combination treatment significantly reduced wrinkles, enhanced collagen fiber density and organization, and promoted inflammation clearance [32]. However, this administration method is somewhat cumbersome and carries a risk of infection. Further research is necessary to optimize EV therapy, not only in the areas of isolation methods and engineering but also in determining the optimal dose, timing, administration, and frequency of EV therapy [181]. Maintaining a sustained, effective EV concentration is crucial for effective wound healing [181].

To preserve the biological activity of EVs and control their release during long-term damage repair, the use of biocompatible biomaterials or biological devices for EV delivery has been extensively explored [182]. MNs emerge as a promising strategy for efficient EV delivery, combining minimally invasive and intelligent modulation properties with the pro-angiogenic, pro-tissue regeneration, and anti-inflammatory properties of EVs, presenting a promising avenue for therapeutic applications [13,183,184].

### 4.1. In Vivo Study

**Figure 2:**
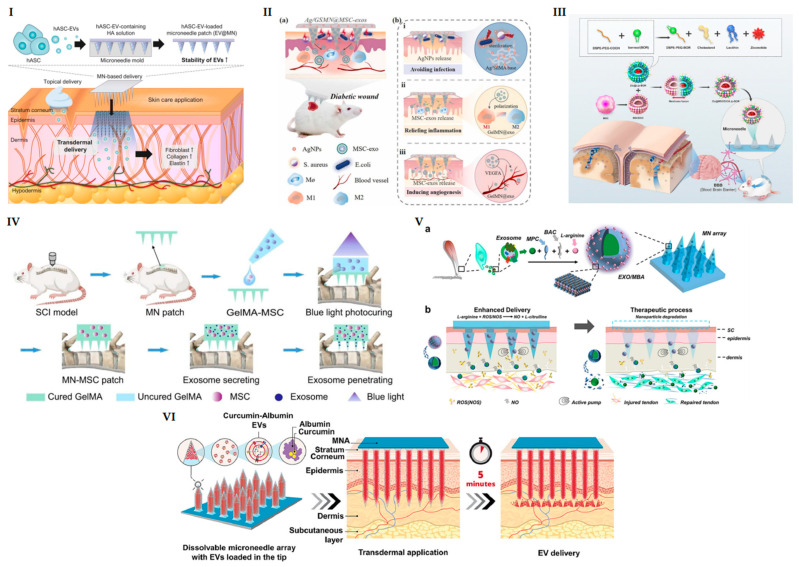
EV-loaded MNs for different therapeutic applications. (**I**) Schematic diagram of the fabrication of EV@MN and skincare applications. Reprinted with permission from [88] (Copyright {2023} Elsevier). (**II**) Illustration of the MSC-EV loaded MN patch for promoting diabetic wound healing. Reprinted with permission from [185] (Copyright {2023} Elsevier). (**III**) MN-mediated delivery of ziconotide-loaded liposomes fused with EVs for analgesia. Reprinted with permission from [192] (Copyright {2023} Elsevier). (**IV**) Schematic illustration of MN-MSC patch implantation on the injury site of the spinal cord. Reprinted with permission from [179]. (**V**) The manufacturing procedure of EXO is modified by a nitric oxide nanomotor-loaded MN array, and the healing process of Achilles tendinopathy occurs after the application of the MN array. Reprinted with permission from [189] (Copyright {2023} American Chemical Society). (**VI**) Schematic diagram of EV-encapsulated curcumin using dissolvable MN for promoting wound healing. Reprinted with permission from [191] (Copyright {2023} Elsevier).

Notably, EV-loaded MNs have shown effectiveness in various damage models, as illustrated in Figure 2. This approach, through combining human ADSC-derived EVs with hyaluronic acid-solubilizing MNs, ensures sustained release of EVs into the dorsal skin of SKH1 mice, stimulating collagen and elastin synthesis and promoting fibroblast proliferation [88]. These MNs, under mild storage conditions, can maintain the bioactivity of EVs for over six months by proving effective on human dermal fibroblasts and enabling precise transdermal delivery [88]. In a rat diabetic wound model, MSC-derived EVs encapsulated at the tips of porous methacrylate gelatin hydrogel-based MNs were used. This innovative design facilitated the delivery of anti-inflammatory and pro-angiogenic MSC-EVs directly to the wound bed, significantly accelerating wound healing [185]. Furthermore, bilayer MNs incorporating M2 macrophage-derived EVs and polydopamine demonstrated efficacy in enhancing rat diabetic wound repair and healing [186]. In another pivotal study by Fang et al., MSC-EVs and MSCs were encapsulated in porous gelatin methacryloyl material to create an MN patch (approximately 4 mm × 4 mm, containing 45 needles) [179]. Applied to a rat T10 spinal cord injury model, the patch showed a rapid initial release of MSC-EVs within the first four days, whereas the release of MSC-EVs from MSC-inoculated MNs persisted for at least a week, aligning with the optimal treatment window for spinal cord injuries (SCI) [179]. This suggests that strategically designed MNs, with their excellent biocompatibility, can enhance stem cell survival, ensuring sustained and efficient delivery of MSC-EVs [187]. A parallel study demonstrated that EVs derived from human amniotic MSCs, attached to peptide-modified hyaluronic acid hydrogels, and locally administered in spinal cord tissues significantly reduced inducible nitric oxide synthase levels [188]. This intervention improved the inflammatory microenvironment, effectively promoting neural tissue repair and functional recovery [188]. These findings underscore the potential of EV-based therapies in regenerative medicine.

Separable MN patches composed of chitosan lactic acid (CL) and ADSC-derived EVs offer a safe and highly effective strategy for treating hair loss [14]. The inherent antimicrobial properties of CL prevent potential infection, while the sustained release of EVs promotes dermal cell proliferation and growth, thereby aiding hair regeneration [14]. In a similar design, Liu and the team prepared an array of MNs loaded with nitric oxide-modified EVs [189]. Accompanied by local nitric oxide production, these EVs passively accumulate at the injury site, significantly inhibiting the inflammatory response in Achilles tendinopathy rats, promoting tendon cell proliferation, and facilitating healing [189].

Researchers have also utilized EVs as drug carriers to enhance the protection and delivery of therapeutic agents, thereby improving therapeutic efficiency [190]. For instance, curcumin and albumin were encapsulated into EVs, which were then integrated into the tips of dissolvable MN arrays [191]. The results indicated that the encapsulated curcumin exhibited enhanced and prolonged stability, significantly and effectively alleviating skin inflammation in vivo in a mouse model [191]. Additionally, MNs were developed by fusing liposomes with EVs derived from MSCs to efficiently load and deliver ziconotide [192]. This approach offers an alternative administration route to intrathecal injection for ziconotide while also providing an effective drug delivery method for analgesia in multiple mouse pain models [192].

### 4.2. In Clinical Study

EVs can be obtained from various cellular sources, including mesenchymal stem cells, fibroblasts, and macrophages, as well as somatic fluids [193]. However, explicit standards for isolation assays and efficacy assessments are currently lacking [193]. Although there are some limitations related to cell-based strategies due to the low survival rate, immune rejection, and tumor formation, using EVs is a helpful strategy to overcome these challenges [194]. In a first-in-human, phase I clinical trial of healthy volunteer adults, platelet EVs demonstrated safe injection as a potential wound-healing treatment [134]. Despite the absence of formal ratification for clinical use, numerous preclinical trials have substantiated the efficacy and safety of EV therapy in applications related to wound healing and tissue regeneration [195,196,197,198]. EVs present a promising cell-free therapeutic approach, particularly when pretreated and combined with a diverse array of novel materials for loading into MNs [179,199]. This approach enables efficient utilization of EVs and drug-loaded EVs while minimizing spinal cord damage [179]. Polymeric materials, chosen for their reliable biocompatibility and smart response, are commonly utilized in current studies for loading EVs [188,200]. Smart hydrogel-forming microneedle arrays as a technique for transdermal drug delivery with stimuli response show great potential in delivering EVs [201,202].

### 4.3. In Extraction Application

Currently, the extraction of EVs from ISF using MNs is widespread [35,37,203]. MNs obtain EVs by researching the skin of colorectal cancer-induced model mice and patient-derived xenograft models of melanoma, thereby facilitating the early detection of melanoma and colorectal cancer and providing a novel liquid biopsy method with both diagnostic and prognostic value [35,204]. In a notable study, researchers utilized MNs functionalized with anti-CD63 antibodies to co-culture with astrocyte-derived EV suspension and capture CD63^+^ EV subpopulations [136]. The method of MNs extracting EVs is expected to enable parallel, high-throughput isolation of various EV classes, thereby providing a direct analysis of biological fluids [136].

MN delivery of EVs addresses some of the current challenges in storage and delivery, offering a potential platform for the clinical application of EVs. Slow-release MNs loaded with EVs also provide a self-regulating drug delivery system for long-acting formulations [205]. Furthermore, the extraction of EVs from interstitial fluid post-MN acquisition for biomarker detection can effectively contribute to the early diagnosis of diseases [35]. The EV-loaded MN delivery system represents a significant advancement in drug delivery technology [154]. However, more specific testing and development are required in the future to further refine and improve this technology.

## 5. Conclusions

EV-loaded MNs offer a promising strategy for targeted and sustained EV concentration [192]. Extracting EVs through MNs for diagnosis and prognosis shows promise for effectively monitoring and controlling disease progression [206]. However, the impact of MNs’ material, structure, and other design factors on the bioactivity and storage time of loaded EVs requires further investigation. In the future, longer time points and larger animal models should be used for further EV-loaded MN clinical translation to test the optimal time, biosafety, and effectiveness. To promote diffusion and improve delivery efficiency, a precise porous MN structure constructed with two-photon 3D printing technology might have higher precision in controlling the size of the MNs [179]. Up to now, there has been little research on EV-loaded MNs in clinical trials. Although the journey from experimental animal models to clinical patient applications is long and challenging, the importance of EV-loaded MNs in designing efficient delivery systems has become clear. Subsequent research will concentrate on needle-loaded EVs, engineered assembly, functionalized loading, and intelligently regulated drug delivery systems to maintain the biological activity of drugs for more effective, regulated, and targeted delivery.

## Figures and Tables

**Table 1:** Comparison of exosomal isolation techniques.

Mechanism	Techniques	Expertise	Starting Volumes	Time Efficiency	Purity	Cost	Refs
size and density	Ultracentrifugation (UC)	little	100 s of mLs	time- consuming	low	ultracentrifuge high initial cost	[135,139]
	Ultrafiltration (UF)	little	N/A	faster than UC	N/A	minimal equipment	[112]
	Size-exclusion chromatography	N/A	N/A	several hours	purer than UC	requirement high cost	[106]
polymer precipitation	Polyethylene glycol precipitation	little	100 μL to several mLs	quick	low	not expensive instrumentation	[135,140]
	Aqueous two-phase system	easy procedure	N/A	nearly 15 min, yield 10 to 15 times higher than UC	N/A	N/A	[141,142]
immunoaffinity capture	Enzyme-linked immunosorbent assay	common laboratory experiment	100 μl	low	N/A	experiment materials	[137]
	Magneto-Immunoprecipitation	N/A	no up limit	faster than UC	purer than UC	not expensive instrumentation	[137,143]
microfluidics- based methods	Acoustic nano-filter	minimal	50 μL	quick	N/A	N/A	[144]
	Immuno-based microfluidic isolation	minimal	10 s–100 s of μL	rapid	efficient	the most cost	[145,146]

Table abbreviations: N/A denotes not applicable. Refs denote references.

## Data Availability

Data sharing does not apply to this article, as no new data were created or analyzed in this study.
